# Cannabis Derivatives as Ingredients of Functional Foods to Combat the COVID-19 Pandemic

**DOI:** 10.3390/foods14162830

**Published:** 2025-08-15

**Authors:** Xiaoli Qin, Xiai Yang, Yanchun Deng, Litao Guo, Zhimin Li, Xiushi Yang, Chunsheng Hou

**Affiliations:** 1 Institute of Bast Fiber Crops, Chinese Academy of Agricultural Sciences, Changsha 410205, China; qinxiaoli@caas.cn (X.Q.); yangxiai@caas.cn (X.Y.); dengyanchun@caas.cn (Y.D.); guolitao@caas.cn (L.G.); lizhimin@caas.cn (Z.L.); 2 Key Laboratory of Biological and Processing for Bast Fiber Crops, Ministry of Agriculture and Rural Affairs, Changsha 410205, China; 3 Changsha Technology Innovation Center for Plant-Derived Functional Ingredient Exploration and Biosynthesis, Changsha 410205, China

**Keywords:** cannabinoids, COVID-19/SARS-CoV-2, pharmacological and food applications, challenges and prospect

## Abstract

Lower respiratory infections predominantly affect children under five and the elderly, with influenza viruses and respiratory syncytial viruses (including SARS-CoV-2) being the most common pathogens. The COVID-19 pandemic has posed significant global public health challenges. While vaccination remains crucial, its efficacy is limited, highlighting the need for complementary approaches to mitigate immune hyperactivation in severe COVID-19 cases. Medicinal plants like *Cannabis sativa* show therapeutic potential, with over 85% of SARS-CoV-2-infected patients in China receiving traditional herbal treatments. This review explores the antiviral applications of cannabis and its bioactive compounds, particularly against SARS-CoV-2, while evaluating their pharmacological and food industry potential. Cannabis contains over 100 cannabinoids, terpenes, flavonoids, and fatty acids. Cannabinoids may block viral entry, modulate immune responses (e.g., suppressing pro-inflammatory cytokines via CB2/PPARγ activation), and alleviate COVID-19-related psychological stress. There are several challenges with pharmacological and food applications of cannabinoids, including clinical validation of cannabinoids for COVID-19 treatment and optimizing cannabinoid solubility/bioavailability for functional foods. However, rising demand for health-focused products presents market opportunities. Genetic engineering to enhance cannabinoid yields and integrated pharmacological studies are needed to unlock cannabis’s full potential in drug discovery and nutraceuticals. Cannabis-derived compounds hold promise for antiviral therapies and functional ingredients, though further research is essential to ensure safety and efficacy.

## 1. Introduction

Lower respiratory tract infections are more common in children under the age of five and the elderly. Influenza and respiratory syncytial virus (RSV), including SARS-CoV-2, are the most frequent viruses that cause infections with a viral origin [1]. Since 2020, the coronavirus disease 2019 (COVID-19) pandemic has posed a significant danger to worldwide public health. As the COVID-19 pandemic showed, respiratory infections have serious health repercussions. As the number of individuals suffering from respiratory disorders rises, so does the demand for alternative substances that may be utilized to prevent and treat them. Vaccination efficiency is limited due to the virus’s significant genetic diversity. Despite the fact that vaccine development has progressed significantly, a method to reduce immune overactivation in severe COVID-19 patients is still required.

SARS-CoV-2 was initially isolated to China, but it swiftly spread across the world. Since its outbreak, it has killed over six million people all throughout the world. Cases of SARS-CoV-2 are usually asymptomatic or moderate. However, in some patients, SARS-CoV-2 can cause major health problems as a result of cytokine storms, which are defined by an uncontrollable immune response and an overproduction of pro-inflammatory cytokines [2,3].

The use of corticosteroids and hydroxychloroquine, among other drugs, to treat COVID-19 is not always successful. As a result, it is critical to investigate new therapeutic possibilities, such as natural products, for preventing and controlling SARS-CoV-2. Medicinal herbs and natural products have considerable potential in the fight against diseases. More than 85 percent of SARS-CoV-2 infected patients in China have received traditional Chinese medicine treatments, according to a recent publication, and the formulations and natural ingredients employed have recently been examined [4]. Cannabinoids could be one of these choices, considering their anti-inflammatory and antioxidant effects, as well as their ability to maintain the balance between a viral infection and a host’s immune system [2].

**Figure 1:**
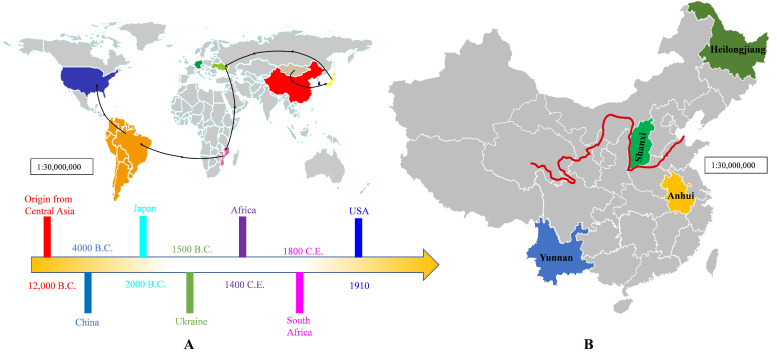
Origin and geographical spread of cannabis species worldwide (**A**), and main production areas in China (**B**). Red line indicates cannabis is cultivated sporadically along the Yellow River [7].

For almost 5000 years, bast fiber crops, such as industry hemp (*Cannabis sativa*) of the *Cannabaceae* family, have been domesticated and widely employed in herbal medicines, textiles, and construction materials [5]. According to their ratios of cannabidiol (CBD) and 9-tetrahydrocannabinol (THC), cannabis crops can be categorized into three subspecies: *Cannabis sativa*, *Cannabis indica*, and *Cannabis ruderalis* [6]. *Cannabis sativa*, sometimes known as hemp or cannabis, is a plant that originated in Central Asia, spread to the Middle East and Europe, and is now extensively dispersed in temperate and tropical locations around the world [7] (Figure 1).

*Cannabis sativa* is a dioecious annual plant that contains a high variety of secondary metabolites including phytocannabinoids, polyphenols, and terpenoids, all of which have antimicrobial, anti-inflammatory, and neuromodulatory properties [8]. These compounds are commonly employed as scents, food additives, and natural pesticides, although they are primarily a source of medications [9]. Cannabis was used to treat various diseases such as inflammatory disorder and malaria since the Han dynasty in China. Cannabis has been used to treat a variety of medical ailments in contemporary times, including pain relief, nausea, and intestinal inflammation [5]. Due to the entourage effect and cannabis’s ability to regulate immunological homeostasis, it has recently been employed as a supplemental therapy for cancer patients [10]. While much is known about cannabis’s neuromodulatory functions, little is known about its potential antiviral mechanisms.

The principal components of cannabis, as well as its antiviral actions and possible application in food, are covered in this review study. This manuscript focuses on the antiviral properties of cannabis and its bioactive constituents and examines how cannabis can be used to combat SARS-CoV-2. This manuscript then reviews the potential applications of cannabis in pharmacology and the food sector. We hope that by conducting this review, we will be able to provide a clearer picture for food manufacturers and researchers of the potential of using hemp polyphenols and terpenes as future functional food ingredients, as well as information on standardizing and improving hemp processing technologies for the efficient production of high-quality, food-safe products. Given the growing interest among scientists on the possible health advantages of cannabinoids derived from *Cannabis sativa* in food production, we want to compile the most up-to-date information on the plant’s features, cannabinoids, and potential for use in food.

## 2. The Pharmacological and Bioactive Constituents in Cannabis

### 2.1. Cannabinoids

**Figure 2:**
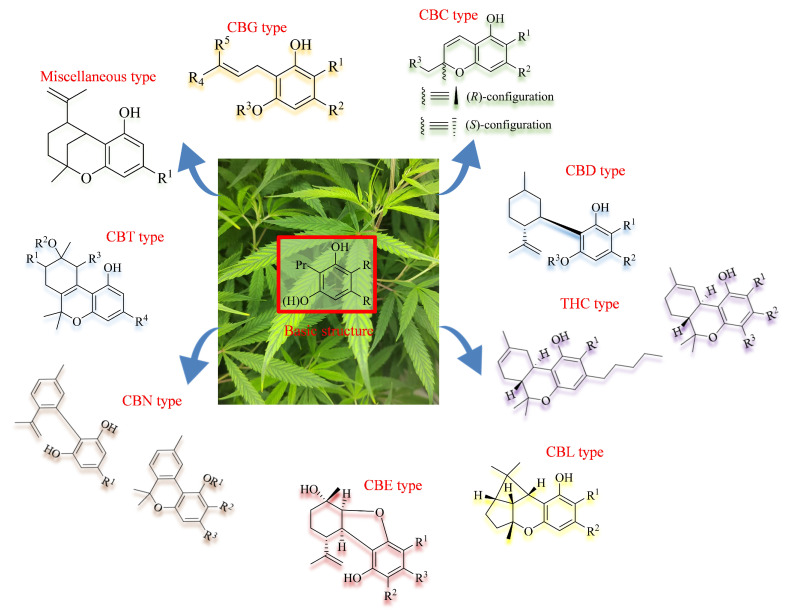
The structures of representative cannabinoids in *Cannabis sativa*.

Cannabis has been farmed for millennia as a source of traditional medicine and textile fiber, but it is now also being recognized as a source of a variety of secondary metabolites with value as medicines, flavoring compounds, and fragrances due to its unique composition and structure [11,12,13]. Cannabinoids are chemical compounds with a wide range of structural diversity. This diversity has been explored relatively extensively due to the strong general interest in cannabis phytochemistry [14]. It was shown that more than 100 different cannabinoids have been isolated from *Cannabis sativa* and they can be classified into about 10 groups, including cannabigerols (CBGs), cannabichromenes (CBCs), cannabidiols (CBDs), Δ^9^-trans-tetrahydrocannabinols (Δ^9^-THCs), Δ^8^-trans-tetrahydrocannabinols (Δ^8^-THCs), cannabicyclols (CBLs), cannabielsoins (CBEs), cannabinols (CBNs), cannabitriols (CBTs), and miscellaneous cannabinoids [15] (Figure 2).

The therapeutic mechanisms of cannabinoids primarily involve interactions with the endocannabinoid system (ECS) through receptor-dependent and independent pathways [16,17]. Receptor-mediated signaling pathways include CB1 receptors (abundant in CNS), which modulate neurotransmitter release (e.g., GABA/glutamate) via Gi/o protein-coupled inhibition of Ca^2+^ channels, regulating pain, mood, and memory [18], and CB2 receptors (predominantly in immune cells), which suppress adenylate cyclase, reducing pro-inflammatory cytokines and exerting immunomodulatory effects [19]. Non-receptor pathways include ion channel modulation, by which CBD activates TRPV4 to induce mitophagy in glioma [20], and enzyme inhibition, by which cannabinoids inhibit FAAH/MAGL, prolonging endogenous anandamide activity [21]. There is a third, biased signaling, which explains why recent cryo-EM studies reveal that CB1-β-arrestin1 complex formation triggers selective anti-depressant/analgesic effects without psychoactivity, mediated by a unique “dual-toggle switch” conformation [18]. In order to understand the interaction mechanisms of cannabinoids with receptors, respectively, the composition and structure of cannabinoids and other phytochemicals are related as follows.

#### 2.1.1. THC Type

THC is the major and most potent psychoactive ingredient in cannabis that causes intoxication. THC was discovered in 1964 and it is found in nature as Δ^9^-tetrahydrocannabinolic acid (THCA), which is produced by decarboxylation of THCA [22]. Δ^9^-THC is a partial agonist with high affinity for CB1 and CB2 receptors, the two G protein-coupled receptors found in many higher vertebrates and mammalian organisms [23]. Tetrahydrocannabivarin (Δ^9^-THCV) is a Δ^9^-THC homologue with an n-propyl (C_3_) side chain and a shorter aliphatic side chain than Δ^9^-THC. The pharmacology of Δ^9^-THC has been well defined as a partial agonist of the CB1 receptor, but Δ^9^-THCV’s pharmacology is a little more complicated [24]. Furthermore, depending on the dose and source species, Δ^9^-THCV has variable affinity for the CB1 receptor. Δ^9^-THCV was proven to decrease the effects of Δ^9^-THC in mice when evaluated in the presence of Δ^9^-THC, but did not appear to behave as an inverse agonist of CB1. However, based on its high binding affinity for CB1 but lack of functional activity, Δ^9^-THCV was found to be a neutral antagonist [25]. Δ^9^-THCV, on the other hand, appears to behave as a partial agonist of the CB2 receptor, with activity being heavily regulated by the level of receptor expression [26].

#### 2.1.2. CBD Type

Cannabidiol (CBD) was separated first in 1940 [27] and its absolute configuration established by synthesis of (-)-CBD as (-)-*trans*-(1*R*,6*R*) [28]. All of the known CBD-type cannabinoids have *trans*-(1*R*,6*R*) absolute configuration and presumably also negative optical rotation. CBD is a non-psychoactive THC isomer that has a variety of pharmacological activities due to its therapeutic potential in a variety of disease states studied in animal models, including pain and spasticity management [29]. Cannabidivarin (CBDV) is a CBD homologue with an n-propyl (C_3_) side chain. CBDV was first isolated from *Cannabis sativa* in 1969 and its optical rotation was reported as [α]_D_-139.5 [30]. In a [^3^H]-20 binding assay in human Sf9 cells, CBDV exhibits a low affinity for the CB1 receptor but a high affinity for CB2 (*K*_i_ = 0.57 μM) [31].

#### 2.1.3. CBG Type

Cannabigerol (CBG) is a monocyclic cannabinoid with a ten-carbon linear geranyl chain and a five-carbon aliphatic side chain. The presence of a linear isoprenyl residue is a structural feature of this molecule. CBG is a non-psychoactive phytocannabinoid that has a low affinity for CB1 and CB2, but has high activity towards many ligand-gated cation channels of the TRP superfamily. It works as a TRPV1 and TRPA1 agonist, as well as a TRPM8 inhibitor. Except for cannabinerolic acid, a recently discovered chemical, there are now seven CBG-type compounds known, all of which have cis-geometry [32]. CBGA is a precursor of CBG, which is used in the biosynthesis of other cannabinoids.

#### 2.1.4. CBC Type

Cannabichromene (CBC) was initially isolated from *Cannabis sativa* in 1966, and it belongs to a special class of cannabinoids with a benzopyran moiety at its core [33]. The benzopyran ring of CBC has a stereocenter, and natural CBC-C_5_ is assumed to be racemic. Furthermore, the C_3_ analog of CBC was isolated [34], and a molecule with a 4-methyl-2-pentenyl side chain at C_2_ was produced, which differed from the 4-methyl-3-pentenyl side chain seen in other CBC-type compounds. The absolute configuration at C_2_, on the other hand, has yet to be defined. CBC has no psychedelic effects in humans. Because its production relies on the decarboxylation of CBCA produced by heating, it is abundant in dried hemp material.

#### 2.1.5. CBL Type

In 1967, CBL was isolated and renamed cannabicyclol/cannabipinol, and the structure was changed [35]. The name THC III was given to CBL because it was thought to have a THC-like structure. Although a [α]_D_-3 was observed [36], CBL from the crude plant material displays no evident optical rotation, and it can emerge as a result of natural irradiation in the plant or as an artifact generated in the crude extract. Using NMR analysis of its methyl ester and a comparison of the decarboxylation product with CBL, cannabicyclolic acid was extracted and identified as the acid of CBL [29].

#### 2.1.6. CBE Type

Cannabielsoin (CBE) is first mentioned in the literature in 1973 [37], albeit no details on its structure are given. The structure and absolute configuration of CBE-C_5_ were finally determined by synthesizing it from cannabidiol diacetate and comparing it to CBE produced by the decarboxylation of natural cannabielsoin acid [38]. Due to the rarity of their detection and/or separation from natural sources, the classification of CBE-type compounds as natural products has been questioned [39]. On various occasions, CBE and CBE acid have been claimed to be natural products of *Cannabis sativa* plant material or hashish [40].

#### 2.1.7. CBN and CBT Type

In the 1930s, cannabinol (CBN) was isolated from hashish for the first time [41]. CBN-type cannabinoids are THC derivatives that have been fully aromatized. Cannabitriol (CBT) was discovered in 1966, and its structure was discovered in 1976 [42].

#### 2.1.8. Other Cannabinoids

Although more than 100 phytocannabinoids have been found in hemp, the majority of them have yet to be completely described [43]. Cannabidivarin (CBDV), cannabivarin, cannabielsoin, cannabicyclol, cannabitriol, and cannabitriol are all terms that are used interchangeably. CBDV is a CBD derivative that varies only in that it has a shorter side chain than cannabidiol. Cannabivarin is a CBN homologue with a shorter side chain, also known as cannabivarol. It is found in modest concentrations in cannabis, rarely detected in new plants, and is primarily found in dried hemp. The oxidation of Δ^9^-THCV results in the formation of this molecule [24,44].

Furthermore, with the rapid advancement of analysis techniques, more and more novel phytocannabinoids are being discovered. In 2019, hemp cannabiphorol (CBDP) and Δ^9^-tetrahydrocannabiphorol (THCP) were identified. These compounds feature seven carbon alkyl chains, making them the first phytocannabinoids with greater than five carbon atoms in a chain, as opposed to most cannabinoid compounds isolated from *Cannabis sativa*. Tetrahydrocannabiphorol has a potential to bind to the CB1 receptor 30 times stronger than Δ^9^-THC, according to in vitro research [45].

### 2.2. Terpenoids

Terpenes are responsible for hemp’s distinctive fragrance and flavor, as well as its protective role in plants. Terpenes have acyclic or cyclic structures that emerge from processes such as reduction, oxidation, cyclization, ring cleavage, or rearrangements within the isoprenoid chain [46]. The monoterpenes pinene, linalool, and limonene, as well as the bitter sesquiterpenes nerolidol, β-caryophyllene, and caryophyllene oxide, have been shown to have antimicrobial, antidepressant, anti-inflammatory, and anxiolytic effects in cannabis essential oils [47]. Geographic location, weather conditions, soil type, fertilizer use, plant age, and weather and time of day or year when cannabis is collected are all elements that determine the essential oil composition.

#### 2.2.1. Monoterpene

Myrcene is the plant’s most significant monoterpene and the smallest terpene in *Cannabis sativa*. It has an unsubstituted acyclic monoterpene with estrogenic action, according to studies. Myrcene activates a highly rectifying conductance that requires the presence of TRPV1 protein to function. Internal calcium levels are extremely sensitive to myrcene-induced currents, and they appear to rapidly inactivate in a manner that is dependent on the degree of calcium buffering in the cytoplasm. Myrcene can be a productive or non-productive ligand for TRPV1 depending on the amount of calcium in the body, which could offer up new avenues for therapeutic treatments with TRPV1 [46].

Myrcene exhibits multiple therapeutic properties. As a key aromatic compound in cannabis terpene profiles, it demonstrates notable analgesic and anti-inflammatory effects by modulating prostaglandin E2 (PGE2) and cytokine pathways, potentially benefiting chronic pain management. Its sedative properties through GABA receptor interaction may improve sleep disorders, while its muscle relaxant effects show promise for spasticity relief. Preclinical studies suggest myrcene enhances cannabinoid absorption by increasing blood–brain barrier permeability, amplifying the entourage effect of THC/CBD. Antimicrobial activity against pathogens like *S. aureus* has been documented in vitro [48,49]. However, current evidence primarily derives from animal models and requires clinical validation.

#### 2.2.2. β-Caryophyllene

β-caryophyllene (BCP), a terpene with a spicy aroma that is easily obtained through heat decarboxylation, is likely the most important sesquiterpene in the cannabis plant. The structure and characteristics of BCP were found to be similar to those of cannabinoid-related compounds. This is the sole terpene that interacts with the body’s endocannabinoid system (binds to the CB2 receptor preferentially). The presence of an oxygen-containing functional group in a molecule generally boosts its antibacterial action, showing a link between structure and biological activity [50].

β-Caryophyllene (BCP), a prominent sesquiterpene in cannabis, exhibits multifaceted therapeutic properties through selective CB2 receptor agonism. Preclinical studies demonstrate its potent anti-inflammatory effects by inhibiting prostaglandin E2 synthesis and modulating cytokines like TNF-α and IL-6, suggesting applications for chronic inflammatory disorders such as arthritis [51]. Notably, BCP promotes white adipose tissue browning via PPARγ upregulation, improving metabolic parameters in obese mice [52]. Its neuroprotective potential is evidenced by oxidative stress reduction and microglial activation suppression, which are relevant for neurodegenerative diseases. Antimicrobial activity against pathogens like *S. aureus* and *E. coli* highlights broad-spectrum defensive capabilities [53].

#### 2.2.3. Limonene

Limonene is a minor terpene component in *Cannabis sativa*, along with cyclic monoterpene. Limonene has antiviral, antibacterial, and antihypertensive effects [54]. It can be found in a variety of essential oil constituents from various plants, which could be due to its precursory involvement in the formation of many monocyclic monoterpenoids. Limonene is the source of the majority of monoterpenes with a 1-*p*-menthene structure, such as carveol, carvone, α-terpineol, pulegone, and 1,8-cineole. Limonene comes in two enantiomeric forms: R and S. It is an optically active chemical. S limonene is commonly found in essential oils of the *Pinus* and *Mentha* species, while R limonene is mostly found in essential oils of citrus peels and by-products [55].

Limonene, a monoterpene abundantly found in cannabis and citrus plants, has garnered significant attention for its diverse therapeutic properties. As a key component of cannabis essential oils, limonene modulates neurotransmitter systems, increasing serotonin and reducing dopamine release to exert neuroprotective and sedative effects [56]. In oncology, preclinical studies highlight its chemopreventive potential in inhibiting tumor proliferation pathways, though clinical evidence remains limited. Pharmacologically, it enhances antibiotic efficacy against resistant bacteria by inhibiting efflux pumps [57]. While existing research primarily derives from in vitro and animal models, human trials are needed to validate these benefits.

### 2.3. Flavonoid

Flavonoids are the most diverse group of polyphenols, with six primary subclasses: flavones, flavonols, flavanones, flavanols, isoflavones, and anthocyanidins. As a by-product of the textile industry, fibrous hemp inflorescences are a source of polyphenol chemicals with proven health-promoting qualities. Flavonoids account for around 10% of the total chemicals found in hemp. Flavonoids can make up around 2.5% of the dry matter in hemp leaves and inflorescences, but only trace amounts are found in the roots and seeds [44]. The O-glycoside aglycone derivatives apigenin, luteolin, orientin, kaempferol, and quercetin, as well as cannflavins A and B, which are methylated isoprenoid flavones specific to hemp, are among the flavonoids isolated from flowers, leaves, and pollen [58]. Despite the fact that flavonoid concentrations vary depending on plant variety, it was discovered that flavone derivatives were most abundant in female inflorescences (apigenin and luteolin). Male inflorescences have less flavonoids than female inflorescences, but they do have two distinct flavonol compounds: quercetin-O-sophoroside and kaempferol-O-phosphoroside.

Cannabis flavonoids, particularly unique compounds like cannflavins (e.g., cannflavin A and B), exhibit significant therapeutic potential. These flavonoids demonstrate potent anti-inflammatory properties by inhibiting prostaglandin E2 synthesis and modulating cytokines like TNF-α and IL-6, offering promise for chronic inflammatory conditions such as arthritis [59,60]. They also contribute to neuroprotection by reducing oxidative stress and enhancing cerebral blood flow, potentially mitigating neurodegenerative diseases like Alzheimer’s disease. Notably, preclinical studies highlight their anticancer effects, where flavonoid derivatives (e.g., FBL-03G) suppress tumor metastasis and synergize with radiotherapy in pancreatic cancer models [61]. Additionally, their antioxidant and antimicrobial activities support cardiovascular health and immune modulation.

### 2.4. Fatty Acids

Hemp seeds have a high content of essential unsaturated fatty acids (about 80% of the total fatty acid content), which gives them a distinctive nutritional value [62]. Hemp seed oil typically has a 2:1 or 3:1 ratio of omega-6 to omega-3 fatty acids, which is considered ideal for human health [63]. Linolenic acid (ALA, 18:3, n-3), which makes up more than half of the total fatty acid composition, is among the remaining fatty acids (LA, 18:2, n-6). Oleic acid (OA, 18:1, n-9), palmitic acid (PA, 16:0), and gamma-linolenic acid (GLA, 18:3, n-6) are also present [64].

## 3. Pharmacological Mechanisms of Cannabinoid Compounds

Cannabinoid compounds have been approved for clinical application based on their plentiful health benefits. As shown in Table 1, several drugs derived from cannabinoids have been used for the treatment of Lennox–Gastaut syndrome, Dravet syndrome, muscle spasticity, etc.

The two most studied phytocannabinoids, Δ9-tetrahydrocannabinol (THC) and cannabidiol (CBD), demonstrate distinct pharmacological profiles (Table 2). Other cannabinoids also show evident pharmacological evidence in vitro or in vivo, although most of them need to be approved clinically. According to the academic focus of this paper, the confirmed and possible pharmacological mechanisms of cannabinoid compounds related to interventions against SARS-CoV-2 are specifically summarized. Based on these mechanisms, the potential of pharmacological applications of cannabinoids in attenuating COVID-19 pandemic is proposed.

### 3.1. Antiviral

Terpenes and terpenoids have a variety of biological and pharmacological properties, including antiviral properties. Compounds from natural sources are of interest as possible sources to regulate viral infection, and medicinal plants provide a variety of chemical elements with the potential to suppress viral replication [64,87]. Since the outbreak of COVID-19, there has been a growing interest in the use of natural chemicals to combat the virus [88]. The ideal therapeutic candidate would be of use for other indications, have a positive safety profile, and have a multitargeted action able to synergistically attenuate cytokine storm while working as an immunomodulatory rather than immunosuppressive medicine [89]. As a result of their activity in modulating the homeostasis between immune response and cell cytokine storm, CBD and other cannabinoids were deemed to be good candidate agents [90,91].

**Figure 3:**
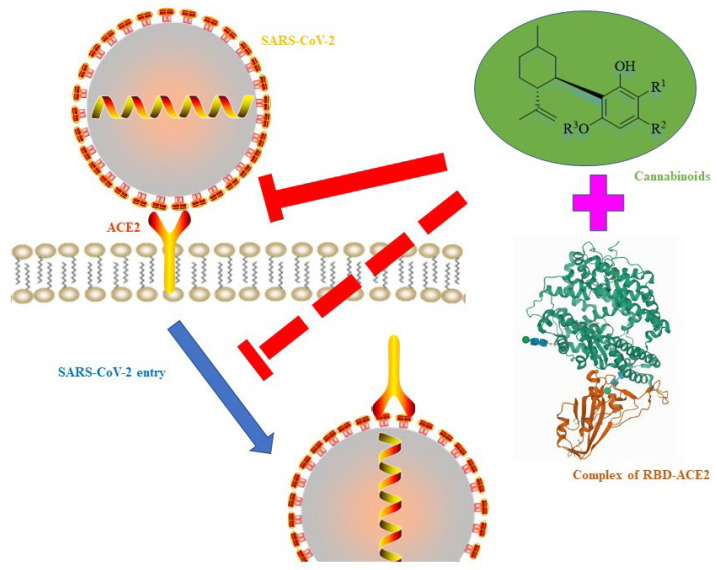
A plausible mechanism of cannabis to inhibit SARS-CoV-2: cannabis blocks the entry of SARS-CoV-2 through binding to a spike protein, ACE2, or complex of RBD-ACE2. The complex of RBD-ACE2 was from the PDB database (ID:7U0N).

The interaction of antiviral medicines with viral proteins is the initial step in their antiviral activity. The most important phase in the viral cycle is the binding of SARS-CoV-2’s spike protein to the human cell surface receptor angiotensin-converting enzyme 2 (ACE2) [90]. By blocking virus particles from invading human cells, cell entry inhibitors could prevent SARS-CoV-2 infection and shorten the course of COVID-19 infections (Figure 3). The expression of transmembrane serine protease 2 enzyme (TMPRSS2) and angiotensin-converting enzyme 2 (ACE2) receptors was reduced in vitro utilizing *Cannabis sativa* extracts rich in CBD with a modest admixture of Δ^9^-THC on human airway epithelium [92]. SARS-CoV-2 enters the host organism through the epithelium of the oral cavity and lungs, using these receptors as entry points [90]. The ability of THC or CBD to bind to the M^pro^ protease of SARS-CoV-2 and limit virus replication has been confirmed [92]. The antiviral effects of this were, however, dose dependent. When the IC_50_ concentration of CBD was 1.86 ± 0.04 µM, it successfully inhibited M^pro^ protease, and when the IC_50_ concentration of CBD was 14.65 ± 0.47 µM, it effectively inhibited ACE2. THC’s IC_50_ inhibition concentration of M^pro^ protease and ACE2 was 16.23 ± 1.71 µM and 11.47 ± 3.60 µM, respectively [93]. CBGA and CBDA were discovered to be allosteric and orthosteric ligands for the SARS-CoV-2 spike protein, and they blocked infection of human epithelial cells by a pseudovirus expressing the spike protein [94]. CBDA and CBGA have recently been found to inhibit infection of the original live SARS-CoV-2 virus as well as variations of concern, such as B.1.1.7 and B.1.351.

Many essential oils include β-caryophyllene, which may play a significant role in their antiviral properties. In vitro, β-caryophyllene has a selectivity index of 140 against herpes simplex virus type 1. The ratio of the cytotoxic concentration of the drug that reduced viable cell numbers by 50% to the antiviral activity that inhibited plaque numbers by 50% relative to the untreated control was used to calculate the selectivity index [64]. Based on a bioinformatic study, it has been found that β-caryophyllene can bind SARS-CoV-2 spike protein and ACE2 [95]. Following this, González-Maldonado et al. [88] discovered that β-caryophyllene had a particular effect in vitro on the SARS-CoV-2 spike-pseudotyped virus. In order to better understand the anti-SARS-CoV-2 capabilities of cannabinoids, Table 3 shows the comparative progress of research on cannabinoids, quercetin, resveratrol, and EGCG against SARS-CoV-2 with key findings from authoritative references.

### 3.2. Immune Regulation

Furthermore, due to phytocannabinoids’ immunosuppressive properties, which can inhibit appropriate antiviral immune responses, vigilance should be exercised [102]. T-cells, B-cells, monocytes, and microglia are all affected by active cannabinoids, resulting in a decrease in pro-inflammatory cytokine expression and an increase in anti-inflammatory cytokines. Almogi-Hazan and Or have examined the role of the endocannabinoid system (ECS), cannabinoid receptors 1 and 2, and cannabinoids in numerous physiological systems, including immunology and diverse diseases [103]. CBD and CBN have the ability to change the immune system’s functioning. CBD, for example, may operate as an immune suppressor by inhibiting the activation of several immune cell types, inducing death, and promoting regulatory cells, which in turn govern the activity of other immune cell targets [104]. The ECS is the glue that holds everything together. Because our bodies naturally have cannabinoid receptors, compounds contained in cannabis are recognized to have potential effects in humans. Our bodies spontaneously create endocannabinoids when they are needed. To induce specific responses, they alter cell activity and travel backward through chemical synapses. The endocannabinoid system, like the immune system, is a foundational function that the body employs to maintain biological homeostasis.

The downregulation of cAMP adenylate cyclase by CBN is known to cause immunosuppression by lowering intracellular levels of cyclic AMP [105]. The CBN-induced drop in cAMP levels inhibits interleukin 2 (IL-2) production and modulates immunosuppression via disrupting the ERK signaling pathway. CBN has been demonstrated to impede AP-1 binding to the promoter region of IL-2, resulting in immune response inhibition [106].

Furthermore, unlike THC, THCA can reduce the amount of TNF-α in culture supernatants from LPS-induced macrophages in a dose-dependent manner. Although these cannabinoids impair cell-mediated and humoral immunity in animal models, as well as resistance to bacterial and viral infections [107], there is no conclusive evidence that they can impair immune function in humans, as measured by the number of T lymphocytes, B lymphocytes, and macrophages, or immunoglobulin levels [108].

### 3.3. Anti-Inflammatory

In the SARS-CoV-2 virus, abnormal cytokine and pro-inflammatory molecule release is linked to lung damage, multiorgan failure, and ultimately poor prognosis [109]. Hemp extracts have long been linked to anti-inflammatory properties. One of the most important features of CBD is its ability to reduce inflammatory responses and protect against acute and chronic inflammation. CBD has a wide spectrum of anti-inflammatory actions, and it can help with acute lung injuries’ unregulated cytokine production [92]. Recent advances in inflammasome research also suggest that cannabinoids’ anti-inflammatory effects may be mediated in part by modifying inflammasome assembly and activity, suggesting that cannabinoids like CBD could be utilized to treat inflammatory illnesses induced by viral infections like COVID-19 [110].

**Figure 4:**
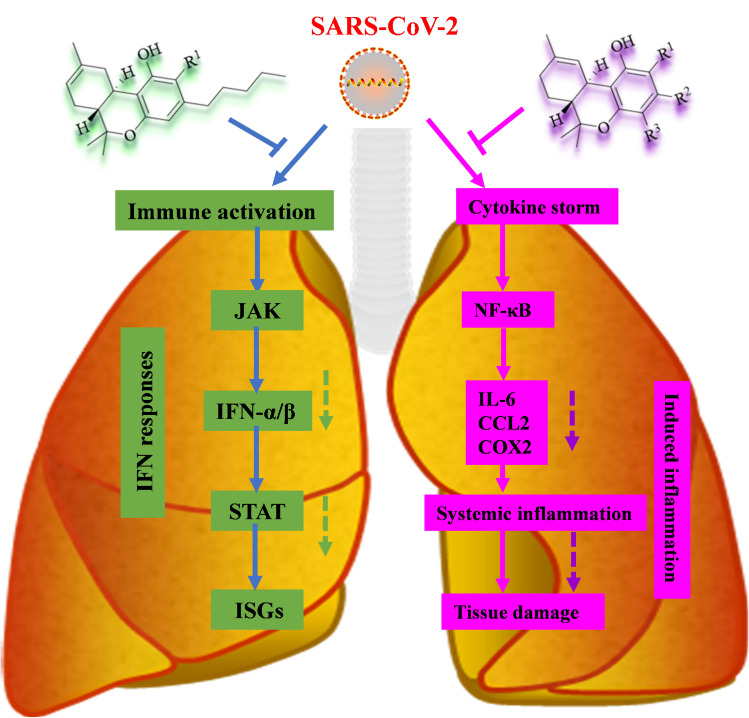
An outline of immune signaling (**left**) and major inflammatory signaling (**right**) is shown, annotated with the known mechanisms by which SARS-CoV-2 activates and suppresses signals (dotted line).

*Cannabis sativa* is well-known for its anti-inflammatory properties, which were previously discussed. COVID-19 causes lung inflammation, which is a serious problem. Inflammation of human lung tissue is triggered by immune responses in severe cases of COVID-19, leading to acute respiratory distress and failure and then causing increased fatality. A cytokine storm is an immunological reaction to the overproduction of pro-inflammatory cytokines [92]. Cannabinoid isolates such as CBD and THC were also studied in humans long before the global pandemic arose as a result of the spread of SARS-CoV-2 infections [103]. CBD, in particular, has demonstrated a strong anti-inflammatory impact via inhibiting CB2 and acting as an agonist on the peroxisome proliferator-activated receptor γ (PPARγ) [2]. This finding suggests that CBD could change host immune response by activating the CB2 receptor, specifically by suppressing inflammation and modulating immunological responses to viral infection (Figure 4).

COVID-19 illness progression is frequently divided into two phases: adaptive immune response and cytokine storm syndrome. Several cytokines, including interleukin (IL)-6 and IL-8, as well as tumor necrosis factor alpha (TNFα), are increased in cytokine storm syndrome. Identified extracts of cannabis with high levels of CBD exhibited anti-inflammatory activity in lung epithelial cells and progressed to induce polarization, phagocytosis, and IL expression in macrophages in vitro, based on the FDA’s approval for the treatment of children with intractable epilepsy for seizure reduction [111]. CBD has been shown to decrease inflammation by inhibiting NLRP3/Caspase-1 response, which is initiated by SP stimulation [112]. CBD has been shown to suppress cytokine storms, protect pulmonary tissues, and restore inflammatory equilibrium [113].

Δ^9^-THCV (0.3 mg/kg and 1.0 mg/kg i.p.) reduced both carrageenan-induced edema and formalin-induced pain in mouse models of acute inflammation and inflammatory pain. When mice were given the CB2 inverse agonist 24, the inflammation was reversed, demonstrating that CB2 regulation plays a role in Δ^9^-THCV’s anti-inflammatory activities [26]. CBDV’s pharmacological effect, on the other hand, could be the result of indirect control of two important endocannabinoid receptors via modification of endocannabinoid processing targets. Because of its high TRPA1 action, CBDV has been studied for anti-inflammatory characteristics in models of irritable bowel disease (IBD) and ulcerative colitis (UC). CBDV was given to animals orally and intraperitoneally at doses ranging from 0.3 to 10 mg/kg before and after dinitrobenzenesulfonic acid-induced colonic inflammation (DNBS). CBDV has been demonstrated to drastically reduce the weight/length ratio of the colon as a result of inflammation and to counteract TRP1A channel overexpression. CDBV inhibited the synthesis of pro-inflammatory cytokines including IL-6 in human colonic tissue taken from biopsies, according to in vitro tests [114].

Furthermore, there were higher levels of cytokines in cannabis smokers’ bronchoalveolar lavage and epithelial brushing, including IL-6, IL-8, TNFα, and IL-10 [115]. Beji et al. reviewed the available data on cannabinoids, viral infections, and the role of mitochondria and came to the conclusion that cannabinoids have the potential to affect a wide range of cell types through mitochondrial modulation, whether through receptor-specific action or not, and that this pathway has potential implications in viral infections [116]. In patients with COVID-19, cannabis smoking or vaping may aggravate cerebrovascular and neurological impairment [117]. Wang et al. [118] recently reported that cannabis extracts suppressed the expression of many inflammatory mediators, including cyclooxygenase-2 (COX2), interleukin-6 (IL-6), and interleukin-8 (IL-8) (IL-8). At this moment, it is unknown whether cannabis extracts or CBD can be utilized to treat any COVID-related health problems [119]. Cannabinoids’ entire antiviral mechanism against SARS-CoV-2 infection is currently unknown. As a result, thorough pharmacological research investigations on the immunotherapeutic potential of cannabinoids against SARS-CoV-2 infection are urgently needed.

### 3.4. Mental Health

Healthcare employees who work with COVID-19 patients are subjected to significant levels of stress, as well as physical and mental exhaustion. Because of their late onset of response and even initial worsening of symptoms, unfavorable motor and cognitive effects, and tendency to cause drowsiness, abstinence, and symptom recurrence after withdrawal, existing drugs are less than optimal against these symptoms [120]. The many unknowns surrounding the COVID-19 pandemic, such as the economy’s state, work opportunities, and a loss of connectivity, might exacerbate sadness, worry, and anxiety. CBD has shown potential as an alternative medication for the treatment of anxiety disorders in clinical trials [104]. CBD’s anxiolytic and anti-depressant effects have led to the suggestion that it could be utilized to treat the mental and physical health of Ebola patients suffering from anxiety and emotional stress [121]. Continuing on the subject of cannabis as a medicine, there have been claims that either smoked or ingested cannabis containing the psychoactive component THC, and those that are natural or synthetic in origin (dronabinol), improves the appetites of people with AIDS, increases weight gain, and lifts mood, thereby improving quality of life.

Serotonin, opioid, and non-endocannabinoid G protein-coupled receptors (GPCR) are all affected by CBD. Because of its anti-inflammatory effects, the medication has immunosuppressive and neuroprotective properties. As a result, it is also involved in CBD’s antidepressant impact and in lowering anxiety disorders including obsessive–compulsive disorder and post-traumatic stress disorder [122]. CBD research has revealed a lot about its anticonvulsant properties, as indicated by the success of CBD in treating epileptic seizures, especially in children. COVID-19 patients, like Ebola patients, may endure a variety of psychological and social difficulties as a result of lingering chronic inflammation and autoimmune reactions. As a result, future randomized clinical trials to assess the efficacy of CBD in reducing anxiety and dread related to COVID-19 infection and its effects on people’s physical, social, and psychological well-being may be beneficial. CBD is most commonly cited as a treatment alternative for anxiety disorders and pain, according to a 2020 study on social media information [123]. CBD has been shown in a recent study to help health care professionals with burnout syndrome symptoms and other mental health issues [124].

Anxiety and post-traumatic stress symptoms (PTSS) or post-traumatic stress disorder (PTSD) linked to the COVID-19 pandemic are anticipated to be a substantial long-term issue. Major calamities, such as epidemics, are known to cause post-traumatic stress disorder (PTSD). According to World Health Organization (WHO) epidemiological forecasts, post-disaster mental health disorders vary from mild to severe distress, affecting 20–50% of a population. Large numbers of persons with long-term anxiety, PTSD, or PTSS are anticipated to be triggered by the present COVID-19 epidemic [125]. According to a more recent survey, the most common reasons for using OTC CBD were stress alleviation, relaxation, and sleep improvement [126].

## 4. Functional Food Applications

CBD and terpene from hemp plants are predicted to be used as future functional food ingredients, receiving the attention of the rising functional foods sector thanks to their great pharmacological and nutritional benefits [127]. Hemp-based foods have a lot of potential to become commercially successful in the functional food market. Some terpenes are GRAS (generally recognized as safe) chemicals that can be added to meals and have been utilized in the past. CBD’s functions, such as pain alleviation, anxiety relief, and nausea relief, have been established in recent studies [128,129]. Consumers are pursuing healthier diets, seeking more functional foods reinforced with functional ingredients, as the prevalence of lifestyle-related chronic diseases rises. Furthermore, because CBD is not psychoactive, it does not have the same addictive properties as THC. As a result, adding CBD or other bioactive chemicals to foods or beverages as future functional food components has a lot of potential [130].

Approximately 74 percent of the US population uses nutritional and dietary supplements, with 55 percent taking them on a regular basis [131]. An appropriate diet is critical for a fully functional immune system and has an early impact on infection risk. Furthermore, malnutrition is common in COVID-19 patients who are elderly [132]. Hemp seeds and hemp-based products have become increasingly popular among consumers in recent years. Hemp seeds, hemp flour, and hemp oil are currently popular culinary products [127]. Cannabis flour obtained from cannabis seeds, as well as cannabinoid oils and/or extracts, are used to make products containing cannabinoids. For example, when hemp flour and protein concentrate were used as natural nutritional and structure-forming agents in gluten-free starch bread, it was discovered that the presence of hemp-based preparations significantly increased the nutritional value of the bread by adding protein, as well as fats, minerals, and dietary fiber in the case of hemp flour. The use of hemp-based formulations, namely protein preparation, resulted in a large increase in bread volume with very minor changes in crumb structure, which is beneficial to both bread makers and consumers [133]. In times of crisis such as floods, earthquakes, wars, and quarantines, various components of the hemp plant and seed can be used as drinks, as a superb nutritional dietary supplement, or as a dried super food. Hemp seed, hemp seed oil, hemp snacks, and hemp protein have all been touted as high-quality, nutritionally complete foods [134]. Hemp sprouts are known for their beneficial cardiovascular and metabolic effects, and they contain more total polyphenols, flavonoids, and flavonols than hemp seeds [135]. According to an EIHA report [136], seed production for food in the United States increased by 92% between 2010 and 2013. Flowers and leaves used in medicine, dietary supplements, and oils production grew by 3000% in 2013 compared to 2010. The worldwide cannabis market was estimated to be worth USD 123.9 billion in 2019. From 2020 to 2027, the market is predicted to increase at a 14.3% annual rate. The rising popularity of these products has resulted in an ever-expanding product line.

The common types of cannabis-infused foods are shown in Table 4, as well as their primary ingredients, key regions where they are legally available, and authoritative references. Data is compiled from high-impact journals (e.g., Nature Communications) and authoritative market reports (e.g., Global Market Monitor), ensuring evidence-based accuracy. Most products derive psychoactive effects from THC or non-psychoactive benefits from CBD, often combined with terpenes for flavor enhancement. Industrial hemp-based foods (e.g., oils) typically contain THC below 0.3% to comply with legal thresholds. Regions reflect current legal frameworks, with North America (Canada, U.S.) and Europe leading in commercialization, while Asia maintains strict prohibitions except for limited medical use.

## 5. Challenges, Regulatory Issues, and Future Perspectives

### 5.1. Challenges and Strategies

Clearly, there are a few obstacles to overcome when it comes to applying cannabinoids to food. The ultimate quantity of these compounds in a finished product is affected by the proportion of each addition, but the content of THC, in particular, must not exceed permitted limits [137]. Furthermore, only one type of cannabinoid, Δ9-THC, is permitted to be used in food, with amounts ranging from 0.02 to 20 ppm [128]. Due to the variability of cannabinoid concentration in plants, using hemp in food processing is particularly difficult. Further research on the food matrices employed and the oils used as carriers is needed to establish the bioavailability of cannabinoids from food [127]. However, additional proof in functional characterization is required for these bioactive chemicals to be utilized in functional meals. Effective hemp CBD incorporation in traditional food products has not been fully explored and published on, which is critical for the creation of future goods. Another issue is the resinous, oily texture of hemp extracts, as well as their solubility in organic solvents, lipids, and alcohols. It is critical to choose the right form (oil, extract) in which to add the cannabinoids to the completed product so that they have acceptable solubility and do not impact the formulation. In most cases, extracts containing Δ9-THC and/or CBD dissolve in edible oils (e.g., coconut or olive). Further processing of these extracts, however, necessitates the creation of an oil-and-water mixture. Understanding how to increase the solubility, stability, and bio-accessibility of terpenes in various food and beverage systems is also a major topic to be answered in order to establish the viability of using hemp terpenes as a future functional food ingredient, similar to CBD.

Tinctures, soft capsules, and beverages are made with oil-water emulsions containing cannabinoids. Surfactants (emulsifiers) such as polysaccharides, proteins, and phospholipids are required for this sort of emulsion. The type of emulsion, oil molecular composition, and ionic strength of the aqueous extract all influence the emulsifier selection. Because hemp extracts are oily and resinous, solid hemp products are difficult to make. To address this issue, support chemicals are utilized to create a lipid matrix that allows for the controlled release of cannabinoids while also preventing their breakdown [128]. Another issue for producers is ensuring concentration homogeneity in each component of the product. Controlling the amount of water in the product and adjusting the packaging (sufficient amount, inaccessibility to oxygen and light) are two strategies that will improve the quality and durability of such products [138]. To determine bioactivity and bioavailability, established methodologies for component quantification and thorough characterization of cannabinoids are also required.

### 5.2. Regulation

Globally, cannabis regulations exhibit significant variation, ranging from full legalization to strict prohibition. Countries like Canada and Uruguay have legalized both recreational and medical cannabis use, with Germany joining in 2024, allowing adult possession (e.g., ≤30 g) and taxed commercial sales. Medical cannabis is permitted in Australia, Brazil, and 37 U.S. states, requiring prescriptions and often capping THC content (e.g., 1% in Thailand). Decriminalization models, as seen in the Netherlands (coffee shops) and Portugal (personal use ≤5 g), tolerate non-commercial possession while restricting public consumption [139]. Conversely, nations like China and Japan enforce strict bans, imposing severe penalties (e.g., 15 year imprisonment in China) [140]. The UNODC notes rising legalization trends but warns of mental health risks from THC, while Prohibition Partners predicts a $55.3 billion market by 2028 [139,141]. Regulatory frameworks continue evolving, balancing public health and economic interests.

### 5.3. Future Perspectives

The cannabis food industry is poised for transformative growth as legalization expands globally and consumer acceptance increases. In the future, the global cannabis food market might demonstrate three significant development vectors. At first, precision dosing technology will revolutionize product standardization. Nano-emulsion techniques now enable much higher bioavailability of cannabinoids compared to traditional edibles [142,143], allowing for predictable onset times and effect durations. Secondly, functional food integration marks a paradigm shift. Leading manufacturers are combining CBD/THC with adaptogens, nootropics, and probiotics to target specific health outcomes. Clinical trials show promising results for sleep-aid formulations (CBD + melatonin) demonstrating evident improvement in sleep latency [144]. And thirdly, regulatory frameworks are driving sophisticated quality control measures. Cannabinoid standards must be made to mandate batch-level cannabinoid profiling and contaminant screening, elevating safety protocols beyond conventional food requirements. Blockchain tracking systems which provide full supply chain transparency from seed to sale should be established. The market bifurcation into recreational and medicinal product lines continues to accelerate. Recreational products emphasize flavor innovation and social experience, with infused beverages capturing a higher percentage of new product launches than before. Medical formulations focus on symptom-specific blends, particularly for chronic pain management [145]. In conclusion, the future of cannabinoid-infused foods will focus on precise dosing, health-conscious formulations, and mainstream market integration while navigating evolving regulations.

## 6. Conclusions

The rising popularity of hemp and its constituents has sparked concerns regarding the safety of cannabinoid-containing nutritional supplements, dried hemp, and food. Many studies on the properties of cannabinoids other than Δ^9^-THC and CBD have opened up new possibilities for the use of cannabinoids in addressing many human health concerns throughout the last decade. Despite this, many functions of cannabinoids found at lower concentrations than the main cannabinoids have yet to be discovered; gaining this knowledge will enable greater utilization of such a diverse set of substances in medicine and functional food production.

Liberalization of hemp production (mostly of low-THC kinds), potential use of hemp plants in the treatment of chronic diseases, and use of hemp as a food additive are all key reasons driving the hemp cultivation market forward. However, incorporating hemp terpenes into foods and preserving terpenes throughout processing are still difficult tasks that require more research. It is also important to figure out if these pathways play a role in SCRA toxicity in humans. Hemp’s potential as a supplementary and healthy food source is still being examined in clinical trials with sufficient power.

## Figures and Tables

**Table 1:** Clinically approved cannabinoid medicines.

Drug Name	Approval Agency	Approval Time	Primary Indication(s) and Mechanism
Epidiolex^®^ (CBD oral solution)	FDA (USA) EMA (EU)	2018 (FDA) 2019 (EMA)	Reduces seizures in Lennox–Gastaut syndrome, Dravet syndrome, and tuberous sclerosis complex-related epilepsy by modulating neurotransmitter release and ion channels
Dronabinol (synthetic THC)	FDA (USA)	1985	Anti-emetic for chemotherapy-induced nausea/vomiting; appetite stimulant for AIDS wasting syndrome
Nabilone (synthetic cannabinoid)	FDA (USA) Health Canada	1985	Treatment of chemotherapy-resistant nausea and vomiting
Sativex ^®^ (THC = 1:1 oromucosal spray)	EMA (EU) Health Canada	2010 (Canada) 2011 (EU)	Relieves muscle spasticity in multiple sclerosis; adjunctive therapy for advanced cancer pain by dual cannabinoid receptor modulation
Cesamet ™ (Nabilone) (synthetic cannabinoid analogue)	FDA (USA) Health Canada	1985 (USA) 1981 (Canada)	Anti-emetic for chemotherapy-induced nausea/vomiting

**Table 2:** Molecular formulas, structural features, medical application statuses of the main 10 cannabinoids.

Cannabinoid Name	Molecular Formula	Structural Features	Medical Applications	Research Status
Δ9-Tetrahydrocannabinol (THC)	C_21_H_30_O_2_	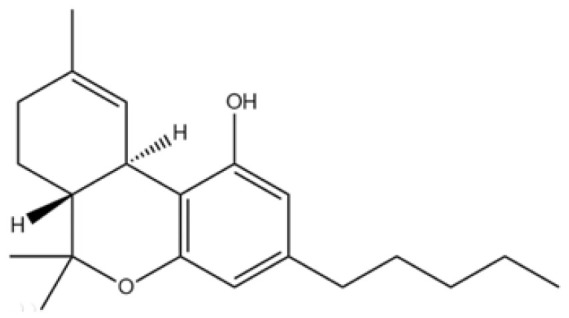	Analgesic [65,66] Antiemetic [67] Appetite stimulation [68]	Clinically synthetic THC (Dronabinol) 1992, FDA
Cannabidiol (CBD)	C_21_H_30_O_2_	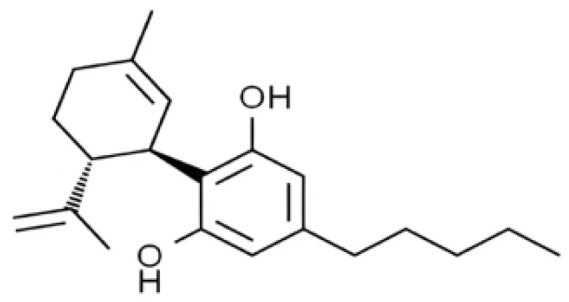	Anticonvulsant [69] Anxiolytic [70] Anti-inflammatory [71]	Clinically CBD oral solution (Epidiolex^®^) 2018, FDA
Cannabigerol (CBG)	C_21_H_32_O_2_	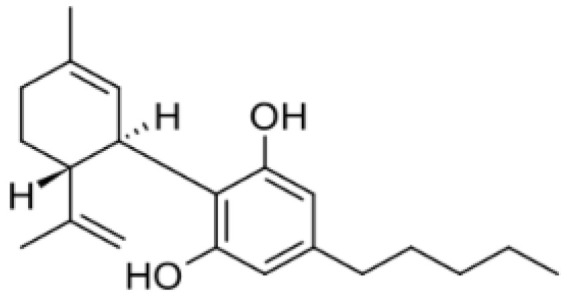	Antibacterial [72] Neuroprotective [73]	In vitro and in vivo In vitro
Cannabichromene (CBC)	C_21_H_30_O_2_	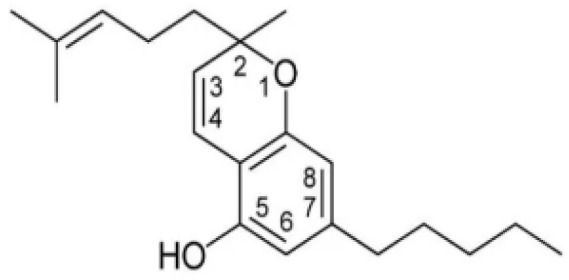	Anti-inflammatory [74] Anti-cancer [75]	In vitro and in vivo
Cannabinol (CBN)	C_21_H_26_O_2_	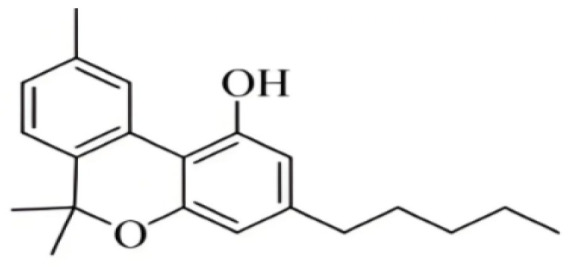	Sedative [76] Anti-oxidant defense [77] Antiaging [78]	In vivo In vitro and in vivo In vitro and in vivo
Tetrahydrocannabivarin (THCV)	C_19_H_26_O_2_	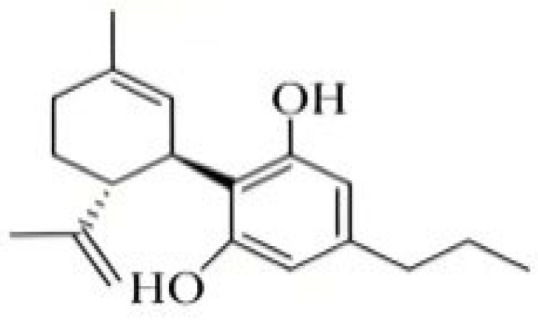	Appetite suppressant [79] Anticonvulsant [80]	In vitro and in vivo
Cannabidivarin (CBDV)	C_19_H_26_O_2_	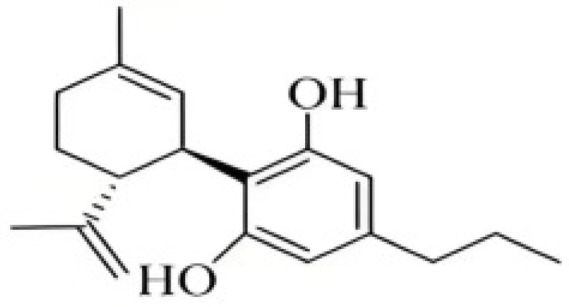	Antiepileptic [81]	In vitro, in vivo, and ongoing-clinically (GW Pharmaceuticals)
Cannabidiolic acid (CBDA)	C_22_H_30_O_4_	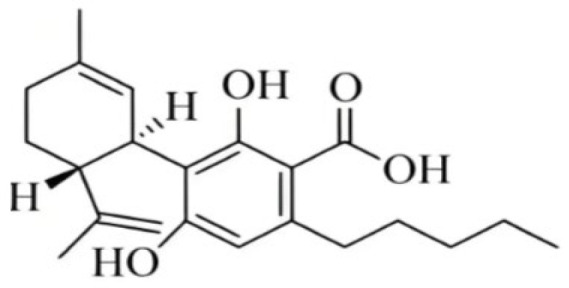	Anti-inflammatory [82] Antiemetic [83]	In vitro and in vivo In vivo
Tetrahydrocannabinolic acid (THCA)	C_22_H_30_O_4_	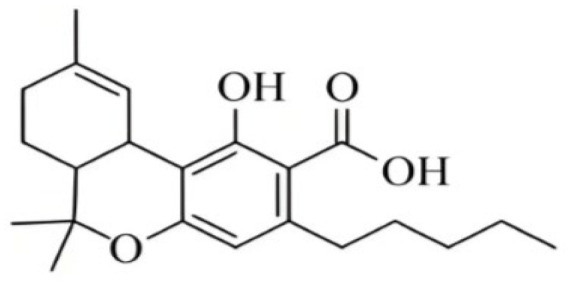	Neuroprotective [84] Antiproliferative [85]	In vivo In vitro
Cannabicyclol (CBL)	C_21_H_30_O_2_	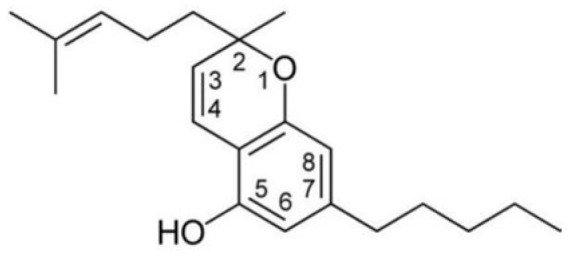	Potential antioxidant [86]	Research ongoing

**Table 3:** The comparative progress of research on cannabinoids, quercetin, resveratrol, and EGCG against SARS-CoV-2.

Compound	Key Anti-COVID-19 Mechanisms	Research Stage
Cannabinoids (e.g., CBD)	Inhibits viral entry by binding spike protein; modulates ACE2 and cytokine storms (e.g., IL-6 reduction) [96,97]	Preclinical/early clinical
Quercetin	Blocks viral proteases (3CLpro, PLpro); stabilizes mast cells to reduce inflammation [98]	Mixed clinical trial results
Resveratrol	Suppresses viral replication via SIRT1 activation; inhibits NLRP3 inflammasome [99]	In vitro and animal models
EGCG	Binds spike protein to block viral entry; modulates TMPRSS2 activity [100,101]	In vitro

Note: All compounds lack large-scale human trials; cannabinoids and EGCG show stronger in vitro antiviral data, while quercetin has more preliminary clinical evidence.

**Table 4:** Main global cannabis-infused food types, key ingredients, regions of availability, and regulatory rules.

Food Type	Key Ingredients	Regions of Availability	Authoritative Rules
Cookies/Candy	THC (≤10 mg/pkg), CBD	Canada, USA, Thailand	Canada: Plain packaging, child-resistant, THC content labels; Thailand: THC ≤ 0.0032% in seasonings
Beverages	THC (≤10 mg/pkg), CBD	Canada, USA	Prohibited with caffeine/alcohol; mandatory THC/CBD labeling
Chocolate	THC, CBD	Canada, USA	Canada: Prohibits health claims; bans appealing designs
Baked Goods	THC (≤10 mg/pkg), Hemp extracts	Canada, Thailand	Thailand: Requires FDA registration for commercial sales
Dietary Supplements	CBD (THC < 0.3%)	USA, Japan	USA: FDA restricts therapeutic claims; Japan: THC-free CBD only
Seasonings/Oils	Hemp seed oil (THC < 0.3%)	Thailand, EU	Thailand: Revised THC limits for non-retail ingredients; EU: Industrial hemp (THC < 0.2%)
Topical Cosmetics	CBD isolates (THC-free)	USA, Canada	China: Bans all cannabis cosmetics; Canada: ≤1000 mg THC per package

## Data Availability

No new data were created or analyzed in this study. Data sharing is not applicable to this article.
